# Lack of Dosage Balance and Incomplete Dosage Compensation in the ZZ/ZW Gila Monster (*Heloderma suspectum*) Revealed by De Novo Genome Assembly

**DOI:** 10.1093/gbe/evae018

**Published:** 2024-02-06

**Authors:** Timothy H Webster, Annika Vannan, Brendan J Pinto, Grant Denbrock, Matheo Morales, Greer A Dolby, Ian T Fiddes, Dale F DeNardo, Melissa A Wilson

**Affiliations:** Department of Anthropology, University of Utah, Salt Lake City, UT, USA; School of Life Sciences, Arizona State University, Tempe, AZ, USA; School of Life Sciences, Arizona State University, Tempe, AZ, USA; School of Life Sciences, Arizona State University, Tempe, AZ, USA; Center for Evolution and Medicine, Arizona State University, Tempe, AZ, USA; Department of Zoology, Milwaukee Public Museum, Milwaukee, WI, USA; School of Life Sciences, Arizona State University, Tempe, AZ, USA; School of Life Sciences, Arizona State University, Tempe, AZ, USA; Department of Genetics, Yale University, New Haven, CT, USA; School of Life Sciences, Arizona State University, Tempe, AZ, USA; Department of Biology, University of Alabama at Birmingham, Birmingham, AL, USA; 10x Genomics, Pleasanton, CA, USA; School of Life Sciences, Arizona State University, Tempe, AZ, USA; School of Life Sciences, Arizona State University, Tempe, AZ, USA; Center for Evolution and Medicine, Arizona State University, Tempe, AZ, USA; Center for Mechanisms of Evolution, Biodesign Institute, Tempe, AZ, USA

**Keywords:** sex chromosome evolution, ZW system, squamate, Anguimorpha, genome assembly, expression, reptiles

## Abstract

Reptiles exhibit a variety of modes of sex determination, including both temperature-dependent and genetic mechanisms. Among those species with genetic sex determination, sex chromosomes of varying heterogamety (XX/XY and ZZ/ZW) have been observed with different degrees of differentiation. Karyotype studies have demonstrated that Gila monsters (*Heloderma suspectum*) have ZZ/ZW sex determination and this system is likely homologous to the ZZ/ZW system in the Komodo dragon (*Varanus komodoensis*), but little else is known about their sex chromosomes. Here, we report the assembly and analysis of the Gila monster genome. We generated a de novo draft genome assembly for a male using 10X Genomics technology. We further generated and analyzed short-read whole genome sequencing and whole transcriptome sequencing data for three males and three females. By comparing female and male genomic data, we identified four putative Z chromosome scaffolds. These putative Z chromosome scaffolds are homologous to Z-linked scaffolds identified in the Komodo dragon. Further, by analyzing RNAseq data, we observed evidence of incomplete dosage compensation between the Gila monster Z chromosome and autosomes and a lack of balance in Z-linked expression between the sexes. In particular, we observe lower expression of the Z in females (ZW) than males (ZZ) on a global basis, though we find evidence suggesting local gene-by-gene compensation. This pattern has been observed in most other ZZ/ZW systems studied to date and may represent a general pattern for female heterogamety in vertebrates.

**Significance**

Squamate reptiles—lizards and snakes—exhibit remarkable variation in sex determination and are thus an important system for understanding sex chromosome evolution. However, because genome sequencing in this clade has lagged behind other major vertebrate groups, major gaps in knowledge remain. We generated a genome assembly for the Gila monster (*Heloderma suspectum*) to better understand its ZZ/ZW system of sex determination. We confirmed previous work showing that Gila monsters share homologous sex chromosomes with Komodo dragons that, if orthologous, date back to the early Cretaceous or late Jurassic. Despite being among the oldest known vertebrate sex chromosomes, we found that males and females have not evolved balanced gene expression on the Z chromosome, which might represent a general pattern for ZZ/ZW systems.

## Introduction

The 11,302 recognized extant species of squamate reptiles, lizards and snakes (Uetz et al. 2021), exhibit remarkable diversity in morphology, ecology, life history, physiology, and behavior (Sites et al. 2011). In particular, modes of sex determination abound in squamates and include temperature-dependent sex determination, male heterogamety (XX/XY sex determination, in which males have an X and a Y and females have two X chromosomes), and female heterogamety (ZZ/ZW genetic sex determination in which females have a Z and a W and males have two Z chromosomes), as well as a combination of multiple modes (Shine et al. 2002; Quinn et al. 2007; Pokorná and Kratochvíl 2009; Gamble et al. 2015; Hill et al. 2018; Pennell et al. 2018; Cornejo-Páramo et al. 2020). The incredible number of transitions in sex determination combined with mosaic patterns of both rapid turnover and relative stasis in the squamate tree make this group ideally suited for understanding aspects of sex chromosome evolution (Gamble et al. 2015, 2017).

Despite this extraordinary diversity, some groups are characterized by stability (e.g. Augstenová, Pensabene, Veselý, et al. 2021). The suborder Anguimorpha contains at least 239 species across seven families (Uetz et al. 2021), yet a series of recent studies suggest that the dominant mode of sex determination in this clade is a ZZ/ZW genetic system, and that sex chromosomes across this clade are likely homologous (Pokorná et al. 2014; Iannucci et al. 2019; Rovatsos et al. 2019; Augstenová, Pensabene, Kratochvíl, et al. 2021; Pinto et al. 2023a). If true, these sex chromosomes are among the oldest and most stable in amniotes (Rovatsos et al. 2019). Dating back to at least 115 to 180 mya (Zheng and Wiens 2016), this system is comparable in age to therian mammals (Wilson and Makova 2009; Graves 2016b).

One important consequence of the evolution of differentiated sex chromosomes from an ancestral autosomal pair is a difference in gene copy number between males and females (e.g. ZZ vs. ZW), leading to an imbalance in gene expression between the sexes. Because deviations from gene dosage balance can have profound and often deleterious phenotypic effects (Birchler et al. 2005), mechanisms are expected to evolve to equalize expression between the sexes (“dosage balance”), and return expression levels on the Z (or X) to those of the ancestral autosome (“dosage compensation”; Gu and Walters 2017). Despite these expectations, dosage balance and compensation are not universal and substantially vary among taxa in both completeness and mechanism (Vicoso and Bachtrog 2009; Graves 2016a; Gu and Walters 2017). Perhaps most striking is the difference between male and female heterogametic systems: dosage balance and/or compensation are often observed in male heterogametic systems (XX/XY), while nearly all female heterogametic (ZZ/ZW) systems studied to date—with the exception of Lepidoptera, a species of brine shrimp, and a larval stage of a schistosome (Walters et al. 2015; Huylmans et al. 2017, 2019; Gu et al. 2019; Picard et al. 2019)—exhibit a lack of dosage balance and incomplete dosage compensation (Mank 2013; Gu and Walters 2017). While putative mechanisms have been proposed to explain this difference (Mank 2013; Mullon et al. 2015), dosage compensation has been studied in very few squamates and work in additional taxa is needed to better understand its evolution (Pinto et al. 2023b).

**Fig. 1.**
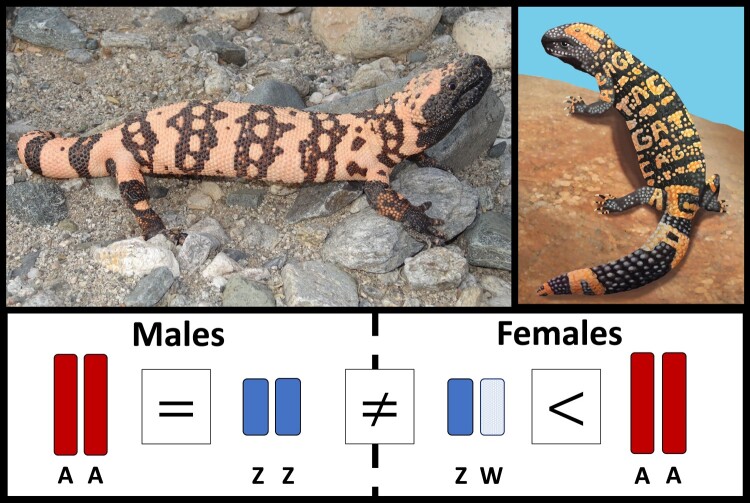
(Top left) The Gila monster, (*Heloderma suspectum*) with its distinctive black and orange pattern, is among the most iconic animals from the deserts of southwestern North America. (Top right) The logo for this project, which started with a crowdfunding effort to assemble a reference genome in collaboration with 10X Genomics. (Bottom) Using DNA and RNA data from six individuals (three males and three females), we investigated Gila monster sex chromosomes (ZW in females and ZZ in males) and their evolution, finding incomplete dosage balance between the sexes and a lack of dosage compensation.

In this study, we sequenced a high-quality de novo genome for the Gila monster (*Heloderma suspectum*; Fig. 1) and generated additional genomic and transcriptomic data from three males and three females to better understand squamate, specifically anguimorph, sex chromosome evolution. Previous studies in anguimorph sex chromosome evolution have identified chicken (*Gallus gallus*) chromosome 28 as the homologous linkage group to the sex chromosome system in varanids, *Abronia*, and helodermatids (Rovatsos et al. 2019). Thus, we (i) investigated whether the ZZ/ZW chromosomes observed across Anguimorpha—the clade containing helodermatids, varanids, and relatives—show evidence of homology at the genomic sequence level, representing a potential single, ancient evolutionary origin with subsequent losses in some lineages (Rovatsos et al. 2019; Pinto et al. 2023a), and (ii) tested for evidence for both dosage compensation and dosage balance in the Gila monster ZZ/ZW system.

## Results and Discussion

### Gila Monster Draft Genome Assembly

We sequenced and assembled a draft genome assembly for the Gila monster (*H. suspectum*) using DNA collected from a wild-born male (ZZ) from Arizona (USA) housed at Arizona State University. The final haploid genome assembly was 2.56 Gb in total length, with a scaffold N50 of 7.86 Mb and a contig N50 of 35.49 kb (supplementary tables S1 and S2, Supplementary Material online). Interestingly, this genome assembly was the largest of any available anguimorph genome, 70%, 25%, and 12% larger than Komodo dragon (*Varanus komodoensis*), Chinese crocodile lizard (*Shinisaurus crocodilurus*), and beaded lizard (*Heloderma charlesbogerti*) assemblies, respectively (Pinto et al. 2023b). The assembled Gila monster genome was 97.2% complete—calculated using kmers—with an average per-base error rate of 7.15316 × 10^−05^ (i.e. <1 error per 10 kb). When estimating genomic completeness in a comparative framework using Benchmarking Universal Single-Copy Orthologs (BUSCO) (v5.1.2) (Simão et al. 2015)—querying two ortholog databases Sauropsida and Core Vertebrate Genes (CVG) (Hara et al. 2015)—we found that our assembly maintains a >90% completeness score. For the Sauropsida database of 7,480 genes, the assembly contains 90.9% complete orthologs, with 1.2% duplicated, 3.4% fragmented, and 5.7% missing. For the CVG database of 233 genes, the assembly contains 94.8% complete orthologs with 0% duplicates, 3.0% fragmented, and 2.2% missing. Thus, the Gila monster genome is largely complete and accurate.

During genome annotation, the Comparative Annotation Toolkit (CAT) identified 15,721 genes in the assembly. 15,129 of these were identified as orthologs of genes in the RefSeq annotation of green anole (18,595 genes). The remaining genes came from comparative Augustus predictions (Stanke et al. 2006), of which there were 131 putatively novel loci, while 617 were predicted to be paralogs, and thus candidates for gene family expansion events. 37 genes had evidence of being split into multiple locations on a single contig, and a further 380 genes had evidence of being split across multiple contigs. To examine the completeness of genome annotation, we again used BUSCO (v5.1.2) (Simão et al. 2015). If the annotation captured most genes present in the genome assembly, the BUSCO scores should be comparable to the unannotated assembly. For the Sauropsida database of 7,480 genes, the annotations contain 75.9% complete orthologs, with 2.5% duplicated, 6.9% fragmented, and 17.2% missing. For the Core Vertebrate Genes (CVG) database of 233 genes, the assembly contains 85.8% complete orthologs with 3.4% duplicates, 7.3% fragmented, and 6.9% missing. Both evaluations of annotation completeness presented much lower scores than that of the full assembly, leaving room for future improvement of the genome annotation.

### Identifying Sex Chromosome Scaffolds in the Gila Monster

**Fig. 2.**
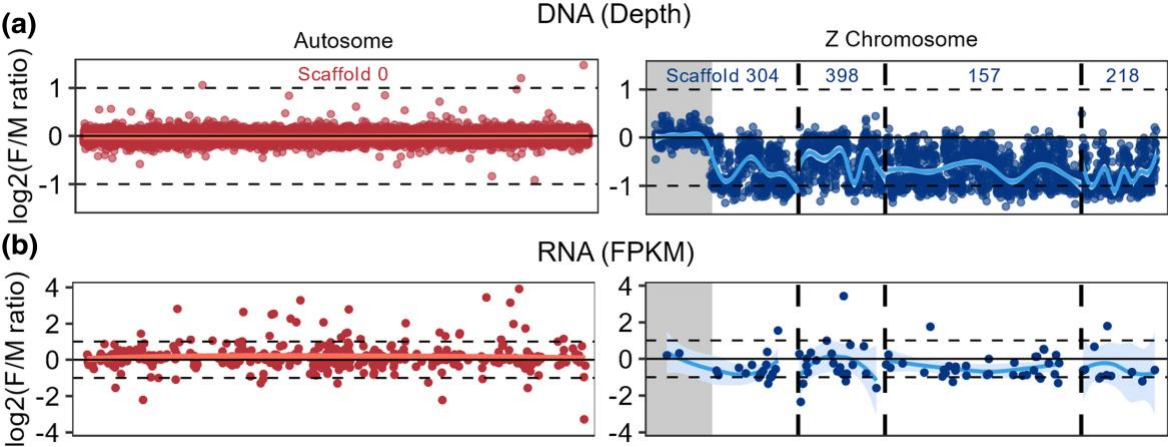
Identifying Gila monster Z-linked scaffolds. Log_2_-transformed ratios of female:male read depth from DNA (A) and female:male FPKM expression for RNA (B) for one autosomal scaffold (0; left) and the four putative Z chromosome scaffolds (304, 157, 218, and 398; right). Log_2_ F/M ratios indicate higher (positive ratio) or lower (negative ratio) read depth and expression in females relative to males. LOESS curves show that read depth and transcript expression are balanced between the sexes for the autosome but vary across scaffolds for the Z chromosome. We ordered scaffolds (separated by dashed lines) in the order in the *S. crocodilurus* genome (304, 389, 157), assigned by RagTag (see Materials and Methods), with the exception of 218, which was not mapped by RagTag and appended on the right end. Our hypothesized pseudoautosomal region on scaffold 304 is displayed in gray. Three female-biased DNA windows on the Z chromosome are not shown on the current plot to make comparable axes across both the Z chromosome and autosome, but were included in statistical analyses: two on scaffold 398 (ratios = 1.79 and 1.78), and one on scaffold 218 (ratio = 2.62). Horizontal dashed lines indicate log_2_-transformed ratios of 1 and −1, while vertical dashed lines in the Z chromosome plots denote the ends of individual scaffolds.

We identified four putative Z-linked scaffolds, greater than 500 kb in length, within the Gila monster genome assembly using mean F/M read depth (Fig. 2; Table 1; supplementary table S3, Supplementary Material online). These four scaffolds (157, 218, 304, and 398) also exhibited an extreme excess of heterozygous sites in females relative to males (supplementary tables S3 and S4, Supplementary Material online). While male heterozygosity (number of heterozygous sites divided by the total scaffold length) on these Z scaffolds overlapped with autosomal heterozygosity, the average number of heterozygous sites on these scaffolds in females ranged from 13 to 35 times that of males. Though genetic diversity on the sex chromosomes can be affected by a number of processes (Webster and Wilson Sayres 2016; Wilson Sayres 2018), it is unlikely to explain these results for three reasons. First, since Z chromosomes are inherited by both sexes, estimates of diversity should not differ between males and females, unless there was a bias in sample collection. To our knowledge this is not the case, and heterozygosity calculated within the sexes are similar on the autosomes–for example, 0.00025 (males) and 0.00024 (females) on scaffold 0—consistent with male and female samples coming from populations with similar demographic histories. Second, Z/A ratios have a theoretical maximum less than 1.2 (Charlesworth 2009; Corl and Ellegren 2012), an order of magnitude less than the female values observed here. Comparing our largest Z-linked scaffold (157) and autosomal scaffold (0), the Z/A diversity ratio is 0.94 calculated using male samples and 27.26 using female samples. Third, outside of pseudoautosomal regions (PARs), females should not have any heterozygous sites because they possess a single Z.

**Table 1:** Summary statistics for each scaffold used for dosage analyses

Sex	Chromosome type	Overall mean	Overall median	Scaffold	Scaffold mean	Scaffold median
Female	Autosomes	28.35	8.91	0	27.38	8.91
				1	32.92	6.51
				2	30.97	8.91
				3	24.98	10.34
	Z	21.18	10.48	157	27.48	20.05
				218	11.69	5.18
				304	19.27	10.22
				398	17.84	4.03
Male	Autosomes	28.40	8.29	0	26.97	8.59
				1	32.74	5.79
				2	31.78	7.96
				3	25.35	9.79
	Z	30.56	18.56	157	40.67	31.48
				218	22.02	8.04
				304	27.34	20.22
				398	21.98	3.18

Means and medians for 3 males and 3 females are presented as FPKM values after filtering out unexpressed transcripts (FPKM = 0 in either sexes). After filtering, 1,099 and 86 transcripts remained across the autosomal and Z scaffolds, respectively. Transcripts from the putative PAR on Z scaffold 304 were removed prior to the above calculations.

Instead, we argue that the extreme heterozygous rates (fraction of sites with non-reference alleles that are heterozygous) we observe in females are technical artifacts. To explore this further, we called variants in the RNAseq data and observed similar heterozygous rates in males and females (supplementary tables S4 and S5, Supplementary Material online). This is in contrast to exons in the nuclear data, which displayed a pattern similar to or more extreme than that of the whole genome. It is unclear why the use of RNA would reduce heterozygous rates to more realistic values than DNA, and this is something that should be explored in future research. However, the similar heterozygous rates in RNA between males and females, the latter of which should lack heterozygous sites, is consistent with mismapping between gametologs—reads from the female W chromosomes mismapping to the Z—because there is no W chromosome in the assembly (Schield et al. 2019; Webster et al. 2019; Pinto et al. 2022).

To better understand the sex-specificity of these putative Z scaffolds, we examined F/M read depth in 5,000 bp windows (DNA) and per-transcript F/M expression in FPKM (RNA) along the Z chromosome, relative to an autosome (scaffold 0; Fig. 2). Autosomal transcripts varied more than Z transcripts in their F/M expression ratios, though locally estimated scatterplot smoothing (LOESS) curves indicated relatively balanced autosomal expression. The Z scaffolds, on the other hand, displayed consistently negative (male-biased) expression ratios regardless of scaffold location. However, in a 1.75 Mb region at the beginning of scaffold 304, read depth and expression ratios matched those of the autosome (Fig. 2), suggesting a PAR. Interestingly, scaffold 304 mapped most proximally, relative to other sex-linked scaffolds, to crocodile lizard chromosome 7 (supplementary fig. S1, Supplementary Material online). We also highlight one scaffold, 674, which was excluded because of its length (<500 kb) and few annotated transcripts (4) but had a low mean female:male read depth and high heterozygosity in females (supplementary table S3, Supplementary Material online). We also found that one gene on this scaffold also maps to chicken chromosome 28 suggesting it too is likely part of the Z chromosome linkage group in Gila monster.

### Synteny of Z Chromosome Scaffolds in Gila Monster

**Fig. 3.**
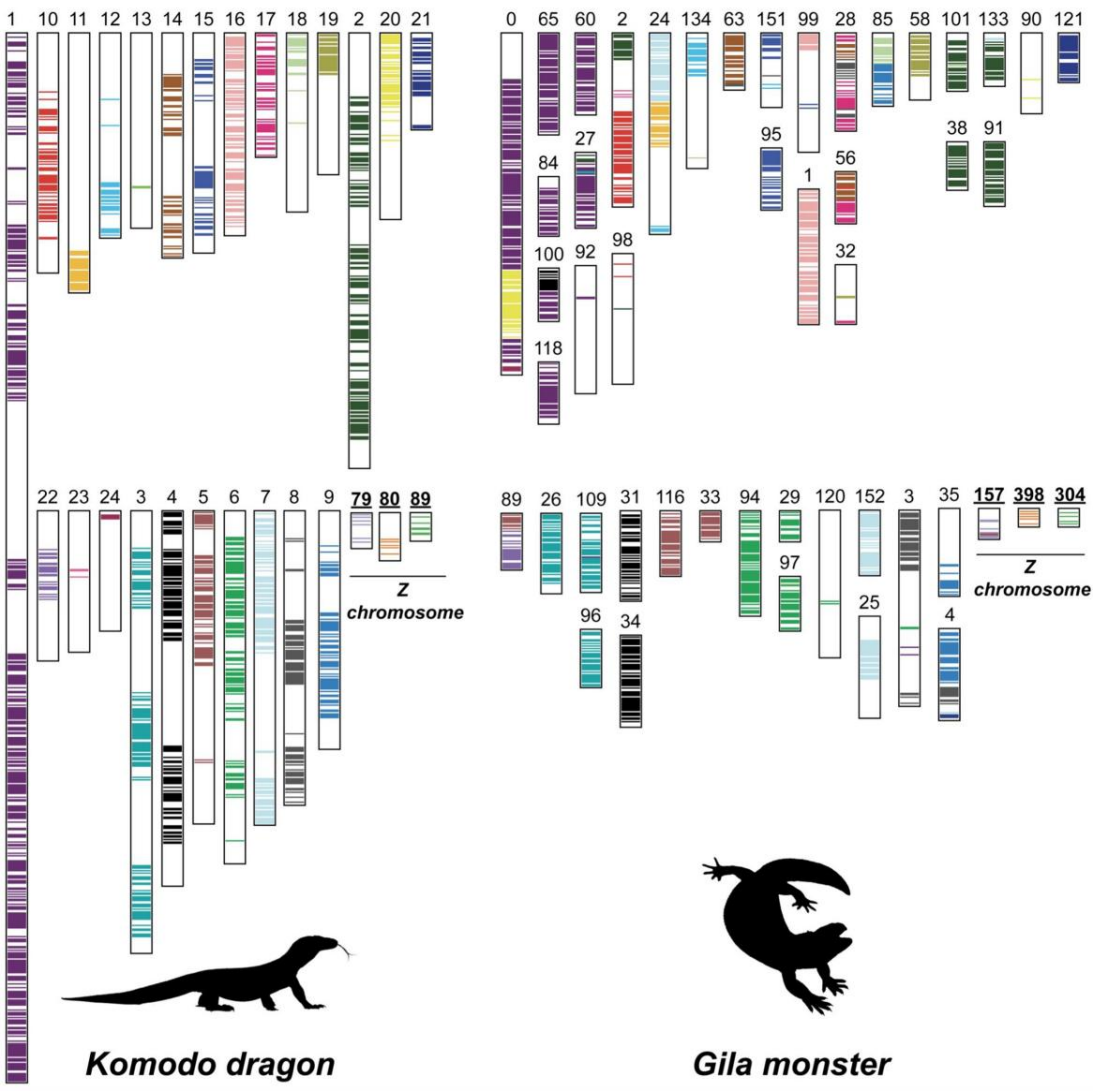
Genome homology between Komodo dragon and Gila monster. For each putative sex chromosome scaffold and the 24 longest scaffolds, we show their position in the Komodo dragon (*V. komodoensis*) genome from Lind et al. (2019) and the Gila monster (*H. suspectum*) genome. Colors indicate homology. Komodo dragon scaffold numbers correspond to their names and annotations on Figshare (Lind 2019), but the three sex chromosome scaffolds are known as 79:SJPD01000091.1, 80:SJPD01000092.1, and 89:SJPD01000101.1 on Ensembl (v105.1).

**Fig. 4.**
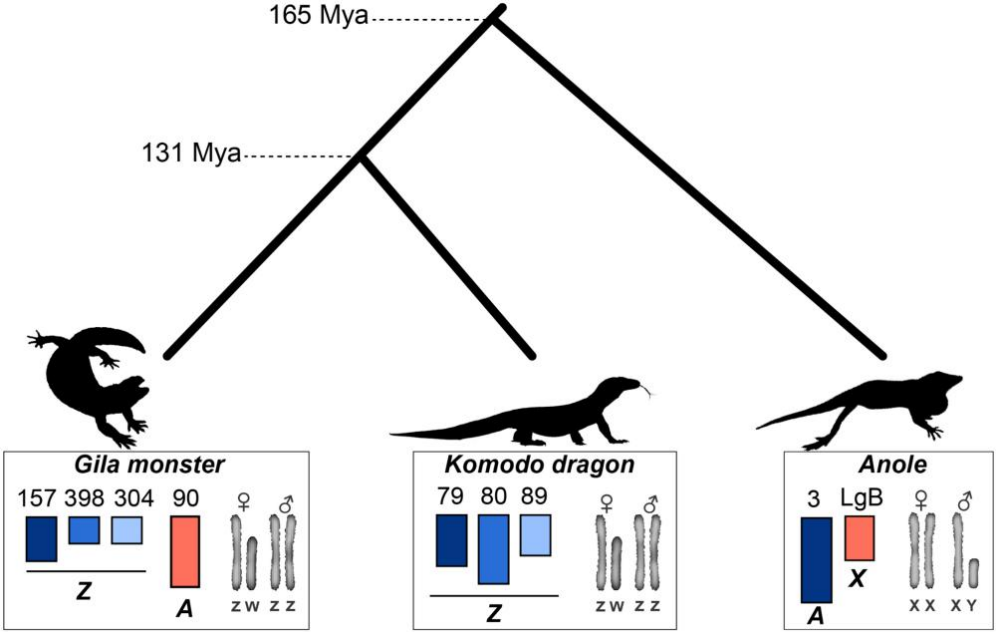
Gila monster sex chromosome sequence similarity with Komodo dragon and green anole. Phylogenetic tree of the relationship among Gila monster (*H. suspectum*), Komodo dragon (*V. komodoensis*), and green anole (*A. carolinensis*), with approximate divergence times from TimeTree.org. Colors indicate homology. The Komodo dragon scaffold numbers correspond to their names and annotations on Figshare (Lind 2019), as described in Fig. 3.

Synteny painting analyses with SynChro (Drillon et al. 2014) revealed that three of the four sex-linked scaffolds in Gila monster are largely syntenic with three corresponding scaffolds in Komodo dragon (Figs. 3 and 4). Further, mapping these scaffolds to the Chinese crocodile lizard genome showed that these sex-linked Gila monster scaffolds (304, 398, and 157) and Komodo dragon scaffolds (SJPD01000091.1, SJPD01000092.1, and SJPD0100101.1) all co-localize to the distal region of chromosome 7 (supplementary fig. S1, Supplementary Material online). Because the fourth Z scaffold, scaffold 218, did not map with SynChro or RagTag, we used LastZ (Harris 2007) to align it with the entire Komodo dragon assembly. The top alignment hit in Komodo dragon was also SJPD01000092.1 (22,510 bp aligned). Therefore, across reptiles, the sex chromosome linkage group in Gila monster and Komodo dragon correspond to chromosome 7 in Chinese crocodile lizard (Anguimorpha), LgB in green anole (Iguania), and chromosome 28 in chicken (Aves).

### Insight Into Sex Determination Mechanisms

We further investigated the functional annotations of genes in the putative Gila monster Z scaffolds. The homologous linkage group in chicken, Gg28, contains the anti-Müllerian hormone (*Amh*) gene, a gene involved in testis differentiation known to act as a master sex determining gene in multiple groups of fishes (M. Li et al. 2015; Myosho et al. 2015; Pan et al. 2019). *Amh* is retained on this linkage group in Gila monster and, in blood tissue, is expressed twice as high in males than females (F/M ratio = 0.513). This linkage group, including *Amh*, is also found on a sex chromosome linkage group in monotremes (Kratochvíl et al. 2021). However, as there are few other potential candidate genes presently assembled on this linkage group, more evidence, (at a minimum) a more complete list of Z-linked genes, is needed to explicitly implicate *Amh* as a candidate primary sex determining gene in Gila monster and other anguimorphs.

### Lack of Dosage Balance With Incomplete Dosage Compensation in Gila Monster

**Fig. 5.**
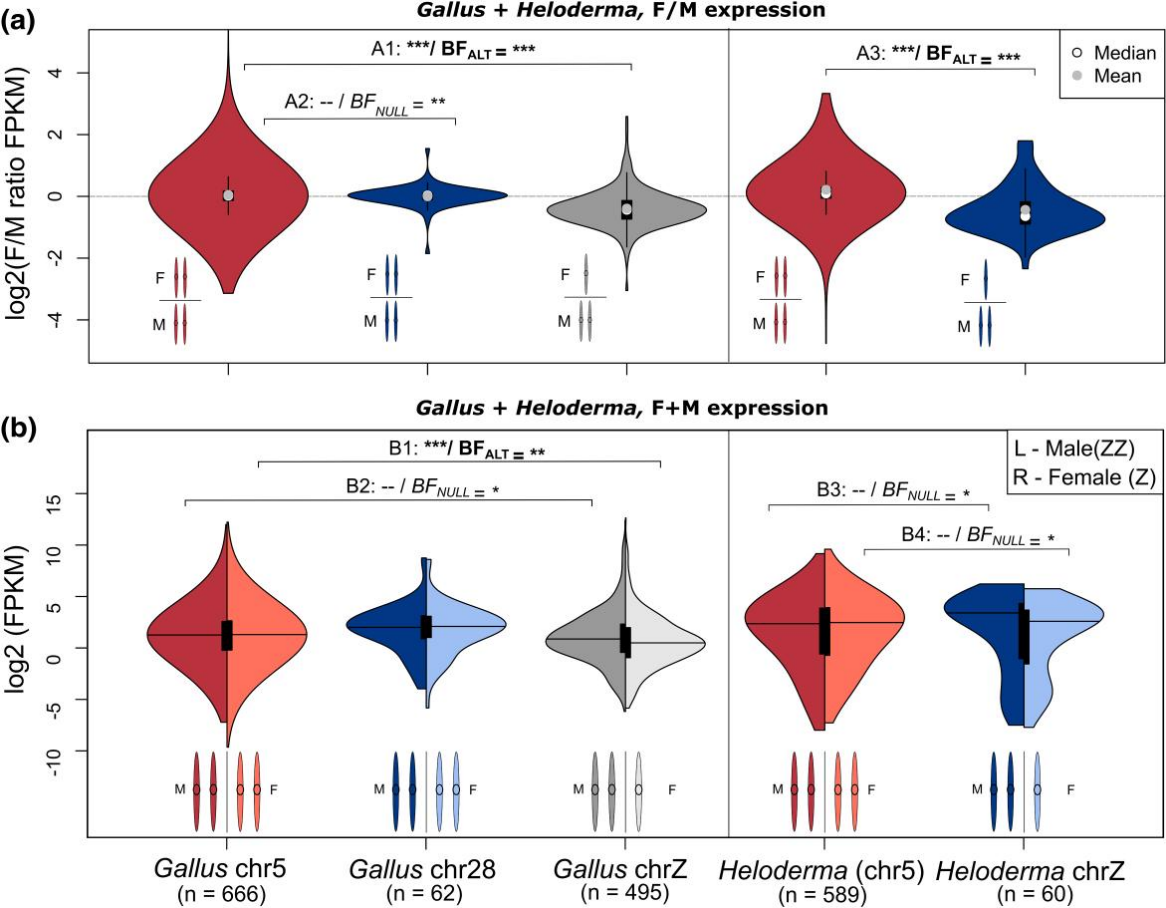
Incomplete dosage compensation without dosage balance in *Heloderma suspectum*. (A) Female/male FPKM transcript ratios across five genomic regions in *Gallus gallus* (chicken) and *Heloderma suspectum* (Gila monster): *Gallus* autosome (chr5), *Gallus* chr28, *Gallus* chrZ, *Heloderma* autosome (syntenic with *Gallus* chr5), and *Heloderma* chrZ (scaffolds 157, 218, 304—excluding putative pseudoautosomal region, and 398). The genomic regions *Gallus* chr5/*Heloderma* autosomal (shaded red) and *Gallus* chr28/*Heloderma* Z (shaded blue) are syntenic. (B) FPKM values separating male (left/darker) and female (right/lighter) violins for each of the five genomic regions. Pairwise statistics comparing each group are Bonferroni-corrected frequentist and Bayesian Wilcoxon rank sum tests. *P*-values and Bayes Factors (Alt/Null) for each test (A1-B4) are reported in the main text. Support values are summarized as ***strong support (*P* ≤ 0.001; BF > 30), **moderate support (0.001 > *P* ≤ 0.01; 30 ≥ BF ≥ 10), *modest support (0.01 > *P* ≤ 0.05; 10 > BF ≥ 1), and “–” for no statistical support. Each pair of support values is presented as “*P*-value support/Bayes Factor support”.

To initially test for dosage balance in Gila monster, we isolated autosomal and sex-linked gene expression data in chicken and Gila monster and used Mann–Whitney–Wilcoxon tests in frequentist and Bayesian frameworks. Frequentist statistics are most commonly used in this scenario, however, Bayesian inference can help provide a more nuanced picture (i.e. show support for the *NULL* and *ALT* hypothesis with varying thresholds, where from Bayes Factors (BF) > 30 are considered strong support to 10 > BF > 1 are considered modest support). To make comparisons between chicken and Gila monster data, given differences in assembly quality and annotation, we selected representative scaffolds that possessed similar numbers of genes, Gg5 (autosome) and Gg28 (sex chromosome). The number of universally expressed transcripts (expressed in both sexes) present on the representative autosome (Gg5) in chicken and Gila monster was 666 and 589 transcripts, respectively. We filtered to include only expressed transcripts with 1:1 orthologs on the syntenic chicken chromosome 28 and Gila monster Z leaving 62 and 60 transcripts, respectively (supplementary table S5, Supplementary Material online). Lastly, there were 495 transcripts expressed on the chicken Z chromosome. We used these data to test for dosage balance between the sexes and found that F/M gene expression was lower on the Z chromosome in both chicken and Gila monster (Fig. 5A; A1 *P* = 2.73 × 10^−57^ & BF_ALT_ = 2.2 × 10^8^, and A3 *P* = 1.5 × 10^−13^ & BF_ALT_ = 257, respectively). This pattern was previously identified in chicken and replicated here for comparative purposes (supplementary fig. S2, Supplementary Material online; Ellegren et al. 2007; Itoh et al. 2007). The log_2_ ratios of Z chromosome genes in Gila monster (mean = −0.44, median = −0.64) are higher than what is expected with a complete lack of dosage balance (i.e. approximately −1.0) (Schield et al. 2019). The linear modeling approach recapitulated the Mann–Whitney *U* results and also identified a lack of dosage balance in Gila monster (Tables 2a and 3a). The full model, which included sex, Z-linkage, and their interaction as fixed effects, performed best ( ΔAICc ≥ 161.11) and was the only model better than the null model (Table 2a). In this model, the interaction between sex and Z-linkage was the only significant term (male ∗ Z-linked *β* = 0.25), consistent with higher expression on the Z in males than females. Thus, the lower F/M expression on sex chromosomes, relative to autosomes, indicates a state of incomplete dosage balance between the sexes (Gu and Walters 2017).

**Table 2:** Results of model selection

Model	Intercept	Δ AICc	Weight
a) Gila only—dosage balance			
Sex + Z_linkage + Sex ∗ Z_linkage	0.0035	0	1
Null	−0.0124	161.11	0
Sex	−0.0033	162.67	0
Z_linkage	−0.0144	163.05	0
Sex + Z_linkage	−0.0053	164.61	0
b) Gila controlling for ancestral expression (chicken)—dosage compensation			
Sex + Z_linkage + Sex ∗ Z_linkage	0.0387	0	1
Null	0.0151	229.35	0
Z_linkage	0.0188	232.35	0
Sex	0.0236	232.85	0
Sex + Z_linkage	0.0273	233.85	0
c) Gila controlling for ancestral expression (anole)—dosage compensation			
Sex + Z_linkage + Sex ∗ Z_linkage	−0.00136	0	1
Null	−0.03125	50.94	0
Sex	−0.01508	53.12	0
Z_linkage	−0.02215	53.54	0
Sex + Z_linkage	−0.00602	53.72	0

The outcome variable was expression (normalized FPKM) for all models. (a) “Gila only” models included individual (1 | individual) and transcript (1 | transcript) IDs as random effects. (b and c) Both sets of models controlling for ancestral expression included those variables along with an interaction between male and female expression in the outgroup (1 | anc_male:anc_female) as an additional random effect. Within each cluster, models are ordered by ΔAICc values, with the best model listed first.

**Table 3:** Results of best linear mixed models evaluating (a) dosage balance and (b and c) dosage compensation

Fixed effect	Estimate	Std. error	*P*
a) Gila only—dosage balance—full model			
Intercept	0	0.04	0.919
Sex (male)	−0.04	0.03	0.178
Z-linkage (Z)	−0.10	0.11	0.379
Sex (male) × Z-linkage (Z)	0.25	0.02	<0.001***
b) Gila controlling for ancestral expression (chicken)—dosage compensation—full model			
Intercept	0.04	0.04	0.335
Sex (male)	−0.04	0.02	0.085
Z-linkage (Z)	−0.2	0.14	0.142
Sex (male) × Z-linkage (Z)	0.32	0.02	<0.001***
c) Gila controlling for ancestral expression (anole)—dosage compensation—full model			
Intercept	0	0.05	0.997
Sex (male)	−0.04	0.02	0.057
Z-linkage (Z)	−0.47	0.26	0.074
Sex (male) × Z-linkage (Z)	0.32	0.04	<0.001***

In all three cases, the full model had the lowest AICc value and was thus considered to be the best model (Table 2). Significant *P*-values indicated by ***.

We first attempted to diagnose dosage compensation status in the Gila monster using Mann–Whitney *U* tests, the most common approach for this problem (Gu and Walters 2017), and the combination of (i) a Z-to-autosome comparison within Gila monster and (ii) an ancestral state comparison proxied by comparing the syntenic linkage group (Gg28) to other autosomes in chicken. We found no significant differences in within-sex Z-to-autosome expression between male and female Gila monster (Fig. 5B; B3: males, *P* = 0.851 & BF_NULL_ = 7.87, and B4: females, *P* = 0.157 & BF_NULL_ = 4.69), a pattern distinct from chicken, which lacks global dosage compensation (Fig. 5B; B1: females, *P* = 3.53 × 10^−7^ & BF_ALT_ = 13.86, and B2: males, *P* = 0.027 & BF_NULL_ = 4.48). Further, we found no sex-biased expression patterns in our ancestral proxy, chicken chromosome 28, relative to autosomes (Fig. 5A; *P* = 0.783 & BF_NULL_ = 12.29). Equal expression between the Z chromosome and autosomes for both males (ZZ) and females (Z) suggested complete dosage compensation on the Z chromosome in Gila monster. There is little evidence for this pattern (Type IV: complete dosage compensation without balance) in nature (Gu and Walters 2017), which suggested that our results might be driven by a statistical artifact. Possible explanations include a small sample size (only ∼60 Z-linked transcripts in Gila monster with orthologs in chicken), variance in expression among transcripts, and differences in expression among individuals. Thus, traditional statistical examinations of dosage compensation may have been underpowered to resolve the dosage compensation status in this system.

As with dosage balance above, we reanalyzed these data in a linear mixed model (LMM) framework as proposed by Walters and colleagues (Walters et al. 2015; Gu and Walters 2017), in which we can account for variation among transcripts and individuals. Using chicken as our outgroup and ancestral proxy, our best model was the full model ( ΔAICc ≥ 229.35), in which the interaction between sex and Z-linkage was the only significant term (male ∗ Z-linked *β* = 0.32; Tables 2b and 3b; supplementary fig. S3, Supplementary Material online). Replacing chicken with green anole produced the same qualitative results, confirming that choice of outgroup did not affect our analyses (Tables 2c and 3c). These results are consistent with incomplete dosage compensation, as Gila monster Z chromosome expression in females remained lower than that of males while controlling for ancestral expression. As this linear model approach both replicated results obtained by our traditional statistical approaches and extended beyond their limitations, we strongly recommend this approach for future studies of dosage compensation.

We therefore infer that Gila monsters possess a ZZ/ZW system characterized by a lack of dosage balance and incomplete dosage compensation. This pattern (Type III in Gu and Walters 2017) has been observed in almost every ZZ/ZW system that has been studied, the major exception being Lepidoptera (Mank 2013; Gu and Walters 2017). Previous work has shown that dosage compensation in ZZ/ZW systems can occur on a gene-by-gene basis (Graves 2016a; Gu and Walters 2017). Our data suggest that this is likely the case for the Gila monster as well, as average female Z expression was greater than half that of males and we observed substantial gene-by-gene variation in relative female Z expression, including multiple transcripts with greater female than male expression (Fig. 2b).

Why global dosage compensation would be more important in male heterogametic than female heterogametic systems remains unclear (Naurin et al. 2010; Mank 2013; Gu and Walters 2017; Chen et al. 2020). If Gila monster sex chromosomes date back to at least the early Cretaceous or late Jurassic (>115 mya; Fig. 4), they stand among some of the oldest known vertebrate sex chromosomes and the extant lack of global dosage compensation cannot be explained by “a lack of time for it to have evolved”—as if dosage compensation were an inevitable outcome of sex chromosome evolution. Another explanation that has been proposed involves sexual selection, whereby greater reproductive skew in males could lead to more intense selection on expression (Mank 2013; Mullon et al. 2015). Models suggest that this could lead to rapid global dosage compensation in male heterogametic systems and slower, more mosaic compensation in female heterogametic systems, where only genes under strong selection in females evolving compensation (Mullon et al. 2015). We explored two hypotheses related to this explanation in the Gila monster using BCV, a measure of variability in expression. The assumption underlying our use of BCV is that genes under stronger purifying selection should exhibit less variation among individuals (Mullon et al. 2015). When comparing the sexes, we found evidence of stronger selection on expression in males than females (male mean = −1.53, female mean = 0.87; Wilcoxon signed-rank test *P* < 1.148 × 10^−15^, *V* = 0), a result also observed in chickens (Mullon et al. 2015). However, in contrast to chickens (Mullon et al. 2015), we found no effect of sexually concordant selection or more intense female selection on dosage balance (supplementary table S6, Supplementary Material online; supplementary fig. S4, Supplementary Material online). Thus, the lack of global dosage on the ancient Gila monster Z chromosome remains a mystery and an important avenue of future research.

## Conclusions

Here, we presented the draft genome assembly of the Gila monster, *H. suspectum*, alongside DNA resequencing and RNAseq data for multiple male and female individuals. We identified four scaffolds (>500 kb) with male-biased patterns of read mapping and gene expression (Fig. 2). We confirmed that these scaffolds are syntenic with the Komodo dragon (*V. komodoensis*) Z chromosome and chicken (*G. gallus*) chromosome 28 (Gg28), as shown previously shown (Rovatsos et al. 2019).

We found a patterns of expression consistent with a lack of dosage balance between the male and female Z chromosomes and autosomes (in line with previous data from varanids; Rovatsos et al. 2019) and incomplete dosage compensation between the Z chromosome and their ancestral autosomal pair (Fig. 5). This pattern has been observed in most other ZZ/ZW systems studied to date and may represent a more general pattern for ZZ/ZW systems (Mank 2013; Gu and Walters 2017). Our assembly of the Gila monster genome contained relatively few Z-linked genes and we could not resolve dosage compensation with the nonparametric tests typically used in these analyses. However, a linear modeling approach, in which we were able to account for variation among transcripts and individuals, allowed us to successfully infer the presence of incomplete dosage compensation. We suggest that other researchers consider this approach for similar analyses. Taken together, this work adds to our understanding of sex chromosome evolution in squamates and more generally.

## Materials and Methods

### Samples and Sequencing

We collected whole blood from the caudal vein near the tail base of six healthy, wild-born Gila monsters (*H. suspectum*)—three males and three females. Blood samples for DNA sequencing were collected into 2 mL ethylenediaminetetraacetic acid tubes (supplementary table S7, Supplementary Material online), while blood samples for RNA sequencing were deposited into 1.5 mL tubes containing RNAlater (supplementary table S8, Supplementary Material online). All samples were immediately stored at −80 °C.

We sent all samples to the Yale Center for Genome Analysis (YCGA) for extraction and sequencing. For whole genome resequencing, samples were extracted following the YCGA's standard protocol (Illumina TruSeq kit) and sequenced across two lanes of an Illumina HiSeq 4000 with 2 × 150 bp paired-end sequencing. To minimize batch effects, we split males and females across the two lanes (i.e. 2 males and 1 female on lane 1, and 1 male and 2 females on lane 2). For RNA sequencing, RNA was extracted and prepared via the RiboZero protocol, after which samples were sequenced on a single lane of an Illumina HiSeq 4000 with 2 × 100 bp paired-end sequencing.

### De Novo Reference Genome Assembly

We shipped whole blood from individual 10 (a ZZ male to improve our assembly of the Z chromosome) overnight on dry ice to 10x Genomics, where high molecular-weight genomic DNA was extracted and libraries were barcoded according to the Chromium Genome User Guide (details specified in Weisenfeld et al. 2017). 10x Genomics generated approximately 140 Gb of raw data on an Illumina HiSeq 2500 and used 115 Gb of these data for the assembly generated with Supernova (Weisenfeld et al. 2017).

We calculated reference genome completeness and per-base quality statistics with kmers using merqury (v1.3) (Rhie et al. 2020). We further estimated genome completeness in a comparative framework using BUSCO (v5.1.2) (Simão et al. 2015), implemented on the gVolante web server (v2.0.0) (Nishimura et al. 2017)

### Genome Annotation

Using Cactus (Armstrong et al. 2020), we aligned Gila monster to *Anolis carolinensis* (anoCar2) using garter snake (thaSir1), chicken (galGal5), and frog (xenTro9) as outgroups. The guide tree was “((Chicken:0.437442,(Anolis:0.247,(Gila:0.2,Garter_snake:0.2):0.1)1:0.2)1:0.172,Frog_X._tropicalis:0.347944)”. After alignment, Gila monster was annotated using the Comparative Annotation Toolkit (CAT; Fiddes et al. 2018). To aid the annotation process, we aligned RNAseq from 3 male Gila monsters and passed those alignments to CAT. We also used the RefSeq annotation of *A. carolinensis* as the source annotation set to lift to Gila monster. In addition, we predicted coding loci in all of the species simultaneously with the comparative annotation mode of Augustus (Nachtweide and Stanke 2019).

### DNA Alignment and Variant Calling

We assessed read quality with FastQC (Andrews 2010) and MultiQC (Ewels et al. 2016). We used BBDuk (Bushnell et al. 2017) to remove adapter sequences and trim reads for quality (“ktrim=r k=21 mink=11 hdist=2 tbo tpe qtrim=rl trimq=15 minlen=75 maq=20”). Cleaned reads were mapped to our reference assembly with BWA MEM (Li, 2013) and duplicates were marked with SAMBLASTER (Faust and Hall 2014), before using SAMtools (Li et al. 2009) to fix mates, and sort and index BAM files. We calculated basic BAM statistics using sambamba (Tarasov et al. 2015) (supplementary table S7, Supplementary Material online).

For variant calling, we used GATK4 (Poplin et al. 2018). This multistep process involves first calling variants in each sample separately with HaplotypeCaller (“-ERC GVCF –do-not-run-physical-phasing”), then combining GVCF files from all six individuals with CombineGVCFs, and finally jointly calling variants across all samples with GenotypeGVCFs. To make variant calling more efficient, we divided the genome up into 25 segments of approximately equal size, running each of GATK4's steps on each of these segments in parallel before using BCFtools (Danecek et al. 2021) to concatenate the resulting VCF files. Finally, we filtered variants for mapping quality (MQ ≥ 30), quality by depth (QD > 2), sample depth (FMT/DP ≥ 10), and GQ (FMT/GQ ≥ 30) with BCFtools. MQ and QD are site-wide measures, while DP and GQ filters were applied per sample.

### RNA Mapping and Quantification

We processed RNA reads from blood samples as described above for DNA reads, with the exception that we set “minlen=60” in BBDuk (Bushnell et al. 2017) because the RNA reads were shorter than those from DNA. We mapped reads using HiSat2 (Kim et al. 2019) with default parameters for paired-end reads before sorting reads with SAMtools (H. Li et al. 2009). We calculated basic BAM statistics using sambamba (Tarasov et al. 2015) (supplementary table S8, Supplementary Material online). We next assembled transcripts using StringTie (Pertea et al. 2015) using a reference-based approach.

### Z Chromosome Scaffold Identification

We identified candidate Z chromosome scaffolds using a two-step approach. First, we used the CHROM_STATS module in XYalign (Webster et al. 2019) with the “–use-counts” flag to gather mapped read counts per scaffold. As an approximation of depth of coverage, we divided the read count for each scaffold by the scaffold length and then took the mean of this value for males and females. We then calculated the mean female/male coverage per scaffold. While a number of scaffolds exhibited ratios substantially less than 1, as expected for a ZZ/ZW heterogametic system, values did not clearly separate into distinct Z and autosome clusters. When investigating other metrics across scaffolds, we discovered that five scaffolds, in addition to having some of the lowest female to male depth ratios across all scaffolds, also displayed extraordinarily high heterozygous rates in females (defined as the number of heterozygous sites over the number of non-reference sites). Of these scaffolds, four were longer than 500 kb and had greater than ten transcripts (scaffolds 157, 218, 304, and 398), and for the rest of the manuscript we treat these as candidate Z chromosome scaffolds. For our autosomal comparisons, we used the four largest scaffolds (0, 1, 2, 3), all of which had female:male depth ratios near 1 and exhibited female heterozygous rates that were neither close to 1 nor substantially higher than those of males.

Next, we scanned for PARs on the 4 putative Z scaffolds. For each Z scaffold, along with a representative autosomal scaffold (0), we obtained the log_2_ F/M ratio of DNA read depth in 5,000 bp windows, calculated using XYalign (Webster et al. 2019). We used LOESS curves to visualize ratios by genomic location (Fig. 2A), and manually inspected window depths in possible transition regions. While all Z scaffolds had lower overall read depth for females than males, consistent with expectations for female heterogamety, the first 1,750,000 bp of scaffold 304 showed balanced read depth in both sexes, suggesting a PAR (Fig. 2A).

For transcripts expressed in both sexes, we calculated mean expression values (FPKM) per transcript for each sex and visualized the log_2_(F/M) ratio of expression across the autosomal scaffold (0) and four Z scaffolds (Fig. 2B). For most transcripts, we observed lower expression in females compared to males [negative log_2_(F/M ratios)]; however, some exhibited higher expression in females [positive log_2_(F/M ratios)], including 2 transcripts on the candidate PAR on scaffold 304 (Fig. 2B).

### Synteny Analyses

We used four different methods to identify syntenic regions between the Gila monster and other species. First, we used one-to-one orthologs identified by CAT during annotation to identify “ancestral” Gila monster autosomal and Z genes in chicken (*G. gallus*). As in Rovatsos et al. (2019), all orthologs of Z-linked genes in Gila monster are autosomal in chicken and located on chromosome 28.

Second, to assess synteny conservation, we employed bioinformatic synteny “painting” using a custom Perl script (*Gff2fasta.pl* modified from https://github.com/ISUgenomics/common_scripts), Biopython v1.73 (Cock et al. 2009), and conversion scripts from the CHROnicle package (v2015). We downloaded the genome FASTA and GFF annotation files of Komodo dragon (*V. komodoensis*; Lind et al. 2019) and then extracted and aggregated protein FASTA records using the modified Perl script *gff2fasta.pl*. Using the genome FASTA and a custom Python script longest_scaffolds.py, we identified the 24 longest scaffolds in the Komodo dragon genome. We extracted proteins from these scaffolds and six identified sex chromosome scaffolds from the protein FASTA records using a custom Python script *pull_id_match.py*. This was also performed for the 50 longest scaffolds and five putative sex chromosome scaffolds in Komodo dragon. SynChro computed conserved synteny blocks with delta = 4, which requires four consecutive genes to match across species to be considered a syntenic block (Drillon et al. 2014).

Third, because synteny painting only successfully identified syntenic regions in the Komodo dragon genome for three of the four putative Z chromosome scaffolds in Gila monster, we used LastZ (Harris 2007) to align the remaining scaffold to the entire Komodo dragon genome.

Finally, as we were finalizing this manuscript, the first chromosome-level genome of an anguimorph, the Chinese crocodile lizard (*S. crocodilurus*), was published (Xie et al. 2022). To further resolve the order of scaffolds in Gila monster and Komodo dragon, we mapped these genomes to Chinese crocodile lizard using RagTag (v2.1.0) (Alonge et al. 2021) and visualized them using *pafr* (v0.0.2) (supplementary fig. S1, Supplementary Material online).

### Dosage Compensation and Dosage Balance in Gila Monster and Chicken

In addition to the RNAseq data from three male and three female Gila monsters, we also included chicken (*G. gallus*) and green anole (*A. carolinensis*) as outgroups to approximate ancestral expression. We obtained publicly available RNAseq data from liver tissue for three male and female domestic chickens (Mullon et al. 2015); (NCBI BioProject PRJNA284655; females SRR2889291-3, males SRR2889295-7). Previous analyses confirm that patterns of dosage balance between autosomes and the Z chromosome are consistent across tissues in chicken (Zimmer et al. 2016) and are thus appropriate comparisons to the blood-derived RNAseq data from Gila monsters generated in this study. While we used chicken as the primary outgroup in our analyses because of its better annotation, we also confirmed results using green anole. For this species, we obtained publicly available RNAseq data from tail tissue (NCBI BioProjectPRJNA253971; Hutchins et al. 2014; Rupp et al. 2017; Rupp et al. 2017). Though chicken and green anole differ in sex chromosome complement (ZZ/ZW and XX/XY, respectively), the Gila monster Z chromosome is syntenic with autosomal regions in both species. We processed the chicken and anole data using the same procedures as the Gila monster data (described above). We employed two statistical approaches to evaluate dosage balance and compensation: (i) nonparametric Mann–Whitney–Wilcoxon *U* tests—the most commonly used method for this problem in the literature—and (ii) a linear modeling approach similar to that proposed by Walters et al. (2015) and Gu and Walters (2017).

For our Mann–Whitney–Wilcoxon *U* analyses, we grouped genomic regions as follows: a Gila monster autosomal linkage group (syntenic with chicken chromosome 5) and Gila monster Z chromosome (scaffolds 157, 218, 304 without the putative PAR, and 398); a chicken autosome (chromosome 5), chicken chromosome 28 (syntenic with the Gila monster Z chromosome), and the chicken Z chromosome. To test for dosage balance (within species) and dosage compensation (between species), we compared F/M expression ratios in both chicken and Gila monster (Fig. 5A) and relative expression for each region by sex (Fig. 5B), respectively, using both frequentist (with Bonferroni corrections for multiple testing in each species) and Bayesian Mann–Whitney–Wilcoxon tests using JASP (v0.16.2.0) (Fig. 5; JASP Team 2022). To alleviate confounding effects from potential microchromosome function (Perry et al. 2021), we dissected these expression data further by splitting autosomal genes out by their syntenic position in chicken showing the sex-specificity of the Gila monster Z relative to all other linkage groups (supplementary fig. S2, Supplementary Material online). For comparisons between chicken and Gila monster, we limited analyses to one-to-one orthologs identified by CAT during annotation (see Genome Annotation section above). For our Gila monster-green anole comparison, we separately identified one-to-one orthologs using OrthoFinder (v2.5.4) (Emms and Kelly 2019).

We also tested for dosage balance and compensation using a linear modeling approach, as suggested by Walters and colleagues (Walters et al. 2015; Gu and Walters 2017). It is possible that using means and ratios, as done with the Mann–Whitney–Wilcoxon *U* tests, masks important variation present in the data. In contrast, a LMM allows us to model individual and transcript variation in expression, along with our primary variables of interest. To this end, we fit sets of LMMs to test three conditions: (i) dosage balance, (ii) dosage compensation using chicken as outgroup, and (iii) dosage compensation using green anole as outgroup. We first normalized FPKM values using Ordered Quantile Normalization using the orderNorm transformation, the best supported normalization for the data estimated by the “bestNormalize” package in R (Peterson and Cavanaugh 2020; Peterson 2021). After transformation, we confirmed a normal distribution for the new data using the descdist function in the “fitdistrplus” package (Delignette-Muller and Dutang 2015). For all models, we included transcript ID and individual ID as random effects. In the dosage compensation models, we also included as a random effect the interaction between mean ancestral male and mean ancestral female expression for a given orthologous transcript, measured in the outgroup species. We reasoned that a difference in male and female Z chromosome expression present after controlling for this interaction would indicate divergence from relative ancestral expression and therefore a lack of dosage compensation (Walters et al. 2015; Gu and Walters 2017). For each condition, we started with an intercept-only model and iteratively added sex, Z-linkage, and the interaction between the two as fixed effects. We conducted these analyses in R, using the package “lme4” (Bates et al. 2015) to fit models, MuMIn (Bartoń 2023) for model selection, and sjPlot (Lüdecke 2023) for additional summary functions. We used AICc to determine the best supported model, treating models with ΔAICc of 2 or less as equally supported.

### Sexual Selection and Dosage Balance

We tested the hypothesis that sexual selection might drive the lack of dosage balance across most ZZ/ZW systems following Mullon et al. (2015). Using the Gila monster RNAseq dataset described above, we first obtained read counts per sample per transcript using HTSeq (Putri et al. 2022). We then used edgeR (Robinson et al. 2010) to calculate the biological coefficient of variation (BCV), a measure of variability of expression, for each sex for each transcript and used the log_2_ of BCV for downstream analyses. Highly constrained expression is expected under strong purifying selection, while differences in variability between the sexes are a potential signature of a sex-bias in selection (Romero et al. 2012; Mullon et al. 2015).

Limiting our analyses to the non-PAR Z-linked genes expressed in both sexes that we identified in our dosage balance analyses described above, we tested two hypotheses: (i) selection should be more intense in males as a result of sexual selection stemming from greater variance in reproductive success among males than females, and because of this, (ii) dosage balance on the Z chromosome should occur on a gene-by-gene basis in genes under strong selection in either females or both sexes. We tested the first hypothesis by comparing BCV between the two sexes with a Wilcoxon signed-rank test in R. Because male and female expression tend to be correlated, we ran a PCA to project variability across two orthogonal axes (Mullon et al. 2015). Like Mullon et al. (2015), we found that PC1 corresponded to the intensity of sexually concordant selection, while PC2 represented sex-bias, with greater values indicating a stronger male bias. We used these two variables and their interaction as predictors in a linear model, with log_2_(Female FPKM/Male FPKM) as our outcome.

## Supplementary Material

evae018_Supplementary_Data

## Data Availability

Raw sequencing reads have been deposited in the NCBI SRA under Bioproject PRJNA420754, and the genome assembly is available at GenBank under Bioproject PRJNA1064225. The versions of genome assembly and annotation files used in these analyses have been deposited at Zenodo (Webster et al. 2022). Steps for processing and analyzing RNA and DNA sequencing were built into a Snakemake (Mölder et al. 2021) pipeline, with all software managed via Bioconda (Grüning et al. 2018) in a Conda environment. All code for this pipeline and environment (including software versions) is available on Github: https://github.com/thw17/Gila_sex_chroms. Code used in the synteny painting analyses is available at https://github.com/mmoral31/Gila_Macrosynteny_Pipeline.
