# Supplementary material for: Lack of Dosage Balance and Incomplete Dosage Compensation in the ZZ/ZW Gila Monster (Heloderma suspectum) Revealed by De Novo Genome Assembly
Source: Genome Biol Evol. 2024 Feb 6;16(3):evae018. doi: 10.1093/gbe/evae018 (PMC10950046; doi:10.1093/gbe/evae018)

Supplementary Materials

**Supplemental Table S1: Gila monster (*Heloderma suspectum,* male #10) Genome Statistics, part 1.**	
**Supplemental Table S2: Gila monster (*Heloderma suspectum*, male #10) genome statistics, part 2.**	
**Supplemental Table S3. Per-scaffold depth, expression, and genotype statistics.**	**.xlsx file**
**Supplemental Table S4. Number of variants called and rates of heterozygous sites on representative autosomes and putative Z-linked scaffolds.**	
**Supplemental Table S5: *Heloderma suspectum* and *Gallus gallus* gene expression on Gg28**	**.xlsx file**
**Supplemental Table S6. Results of linear model testing the effect of selection intensity on dosage balance.**	
**Supplemental Table S7: DNA Resequencing Sequencing Statistics for Gila Monster.**	
**Supplemental Table S8: RNA Sequencing Statistics for Gila Monster.**	
**Supplemental Figure S1: *Heloderma suspectum* and *Varanus komodoensis* genomes aligned to the *Shinisaurus crocodilurus* genome.**	
**Supplemental Figure S2: F/M gene expression grouped by chicken chromosomes.**	
**Supplemental Figure S3: Marginal effects of the interaction between sex (ZZ: male; ZW: female) and chromosome type (autosome vs. Z chromosome) on expression in Gila monster.**	
**Supplemental Figure S4. Selection and dosage balance in Gila monster.**	

Supplemental Table S1. Gila monster (*Heloderma suspectum,* male #10) Genome Statistics, part 1.

Description	Statistics
Total Assembly size	2,582,238,107
Total number of scaffolds	80,861
Number of scaffolds greater than 100Kb	566
Maximum scaffold length	60,641,200
Minimum scaffold length	1,000
Scaffold N50	7,855,436
Number of annotated genes	15,721
GC content	44.79
BUSCO Genome (Sauropsida)	C:90.9%[S:89.7%,D:1.2%],F:3.4%,M:5.7%,n:7480
BUSCO Genome (CVG)	C:94.8%[S:94.8%,D:0.0%],F:3.0%,M:2.2%,n:233
BUSCO Annotation (Sauropsida)	C:75.9%[S:73.4%,D:2.5%],F:6.9%,M:17.2%,n:7480
BUSCO Annotation (CVG)	C:85.8%[S:82.4%,D:3.4%],F:7.3%,M:6.9%,n:233

Supplemental Table S2. Gila monster (*Heloderma suspectum*, male #10) genome statistics, part 2.

	**Scaffolds ≥ 1bp**	**Scaffolds ≥ 10kb**	**Scaffolds ≥ 50kb**
Total length	2.58 Gb	2.31 Gb	2.13 Gb
Number of scaffolds	80,861	12,703	913
Number of contigs	152,240	73,769	57,630
Scaffold N50	7.855 Mb	9.23 Mb	9.93 Mb
Scaffold L50	94	78	68
Contig N50	35.49 Kb	42.6 Kb	47.8 Kb
Contig L50	15,710	12,233	10,298
Max scaffold length	60.64 Mb	60.64 Mb	60.64 Mb
Max contig length	469.1 Kb	469.1 Kb	469.1 Kb

Supplemental Table S4. Number of variants called and rates of heterozygous sites on representative autosomes and putative Z-linked scaffolds. Each column corresponds to a scaffold (Z-linked scaffolds with gray background). Within rows, female values are in italics.

	**0**	**1**	**2**	**3**	**157**	**218**	**304**	**398**
**Length[a]**	60641200	30751571	40967282	31118194	6081949	2324804	4438600	2690970
**Variant Count[b]**								
**Males**	19400	7992	12849	8116	1930	654	1882	1174
Females	*20987*	*9384*	*14167*	*9497*	*40000*	*9188*	*20440*	*32441*
**Het Rate DNA[c]**								
**Males**	0.78	0.73	0.75	0.74	0.75	0.79	0.81	0.82
Females	*0.69*	*0.73*	*0.68*	*0.71*	*0.995*	*0.99*	*0.98*	*0.996*
**Het Rate RNA[d]**								
**Males**	0.89	0.91	0.91	0.88	0.86	0.90	0.85	0.88
Females	*0.84*	*0.88*	*0.81*	*0.86*	*0.84*	*0.82*	*0.77*	*0.88*

[a]Total length of scaffold in bp.
[b]Mean (within sexes) number of genotypes with a non-reference allele.
[c]Mean (within sexes) fraction of genotypes with a non-reference allele that are heterozygous (in DNA data).
[d]Mean (within sexes) fraction of genotypes with a non-reference allele that are heterozygous (in RNA data).

Supplemental Table S6. Results of linear model testing the effect of selection intensity on dosage balance. PC1 corresponds to sexually concordant selection (larger values indicate more concordance) and PC2 captures sex-biases in selection intensity (larger values indicate a male bias and smaller values indicate a female bias).

	**Estimate**	**Std. Error**	**P**
Intercept	-0.39	0.09	3.06×10^{-5}***
PC1	-0.11	0.07	0.120
PC2	0.19	0.18	0.292
PC1 x PC2	-0.15	0.14	0.281

Supplemental Table S7: DNA Resequencing Sequencing Statistics for Gila Monster. Read stats calculated using FastQC, mapping stats calculated using sambamba flagstat.

Individual	Sex	Pair	Total Reads	GC Content	Mapping stats (total % mapped/% percent properly paired)
10	Male	R1	130,992,535	46%	99.66%/96.00%
		R2	130,992,535	46%	
16	Male	R1	123,987,146	50%	99.65%/95.94%
		R2	123,987,146	50%	
K01	Male	R1	128,666,741	49%	99.66%/95.98%
		R2	128,666,741	49%	
30	Female	R1	128,299,154	47%	99.60%/95.71%
		R2	128,299,154	47%	
35	Female	R1	127,352,643	49%	98.37%/94.53%
		R2	127,352,643	49%	
L	Female	R1	125,449,468	48%	99.60%/95.73%
		R2	125,449,468	48%	

Supplemental Table S8: RNA Sequencing Statistics for Gila Monster. Read stats calculated using FastQC, mapping stats calculated using sambamba.

Individual	Sex	Pair	Total Reads	GC Content	Mapping stats (total % mapped/% percent properly paired)
10	Male	R1	75,069,664	46%	94.21%/91.54%
		R2	75,069,664	46%	
16	Male	R1	66,659,537	50%	84.35%/80.59%
		R2	66,659,537	50%	
K01	Male	R1	65,347,060	49%	87.04%/82.70%
		R2	65,347,060	49%	
30	Female	R1	61,087,539	47%	91.51%/88.05%
		R2	61,087,539	47%	
35	Female	R1	72,499,762	49%	83.95%/79.45%
		R2	72,499,762	49%	
L	Female	R1	70,669,648	48%	90.28%/86.36%
		R2	70,669,648	48%	

Supplemental Figure S1: *Heloderma suspectum* and *Varanus komodoensis* genomes aligned to the *Shinisaurus crocodilurus* genome. (A) Sex-linked scaffolds in *H. suspectum* mapping to the distal region of *S. crocodilurus* chromosome 7 (LG7). (B) Sex-linked scaffolds in *V. komodoensis* also mapping to the distal region of *S. crocodilurus* LG7. Color schemes change between the whole genome section and the LG7-only section (i.e. color legends on LG7 are specific to that image only).

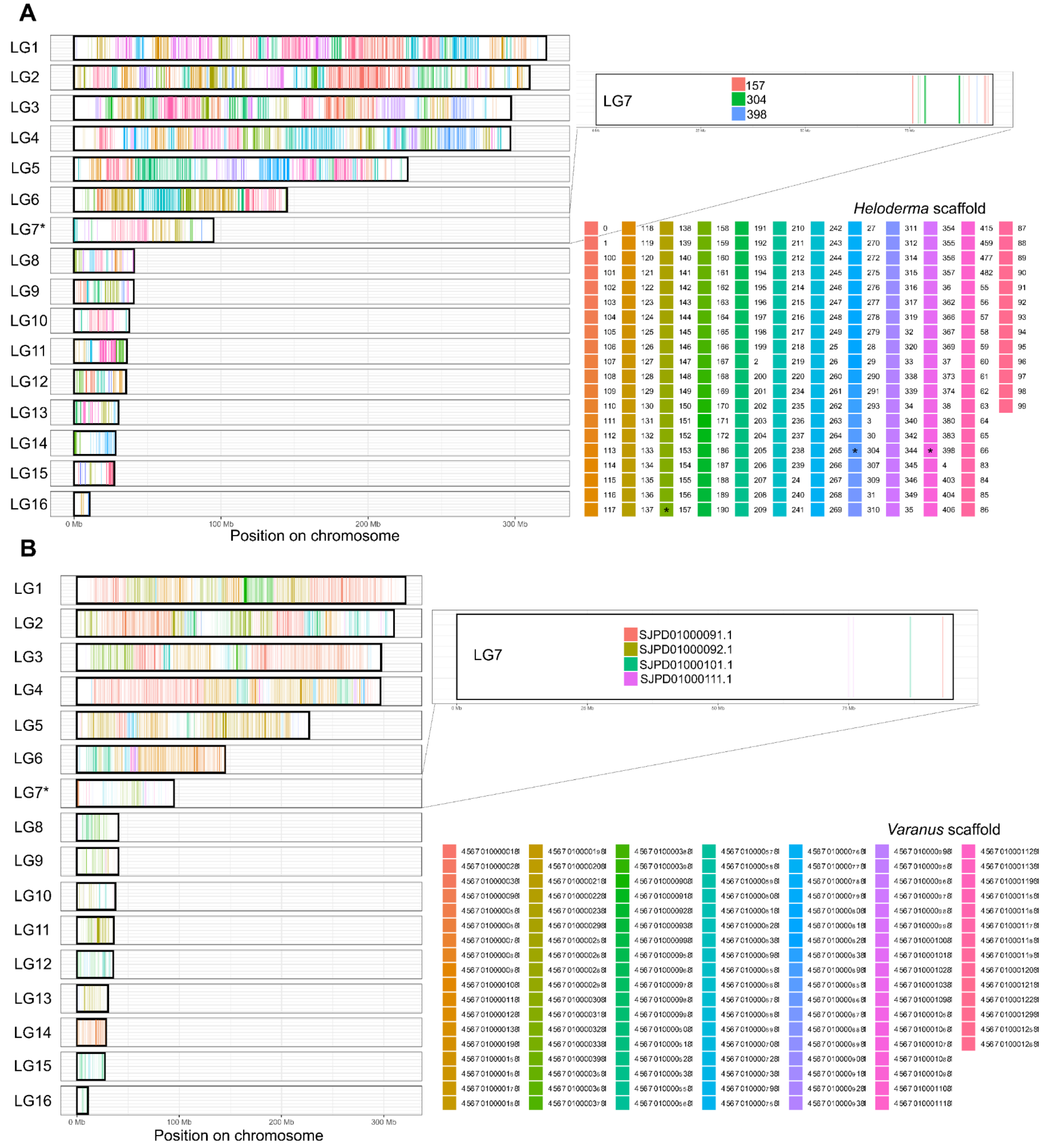

Supplemental Figure S2: F/M gene expression grouped by chicken chromosomes. Log_2(F/M FPKM ratios) for genes clustered by their orthologous position in *Gallus* for *Heloderma suspectum* (top) and *Gallus gallus* (bottom), highlighting the drop in F/M expression in gene orthologous to Gg28 in *Heloderma*.

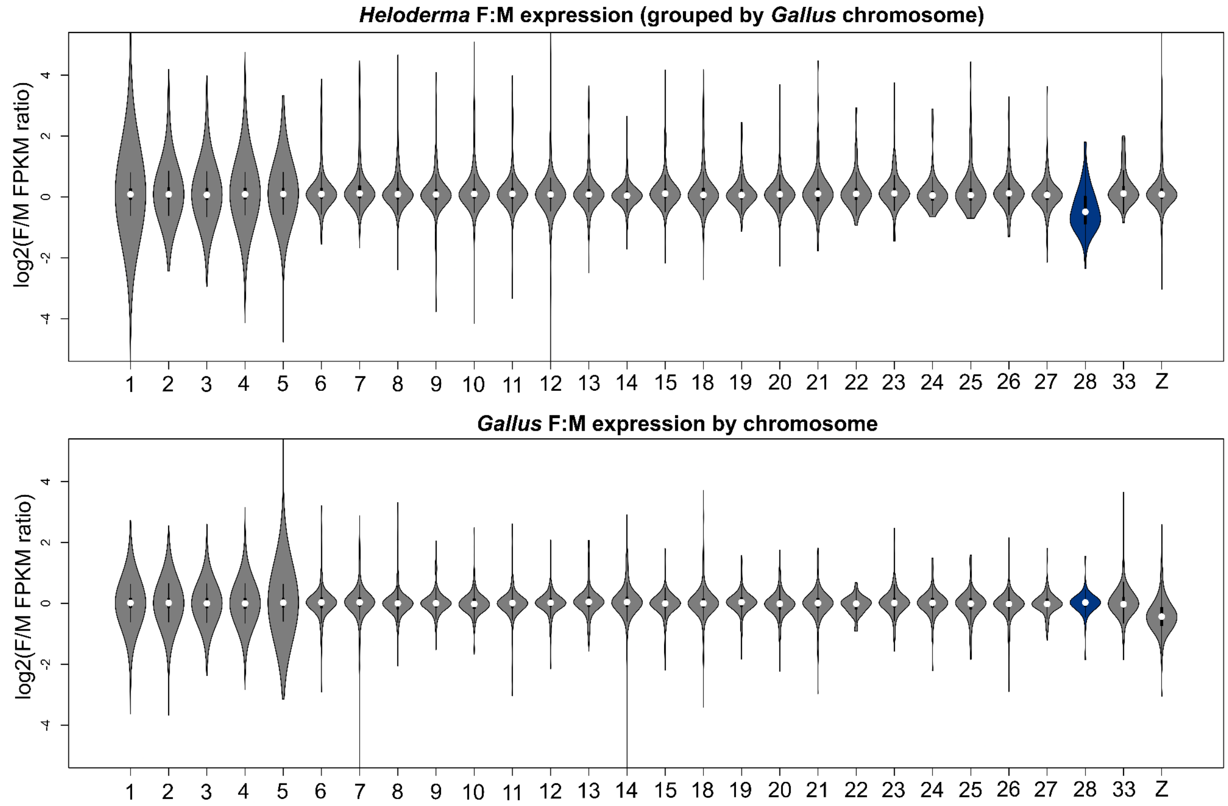

Supplemental Figure S3: Marginal effects of the interaction between sex (ZZ: male; ZW: female) and chromosome type (autosome vs. Z chromosome) on expression in Gila monster. Estimates from the full model for dosage compensation using chicken as an outgroup. Fixed effects were sex, Z-linkage, and their interaction, while transcript ID, individual ID, and the interaction between male and female expression in chicken were included as random effects. Expression in chicken serves as a proxy for expression in the ancestral autosomal condition.

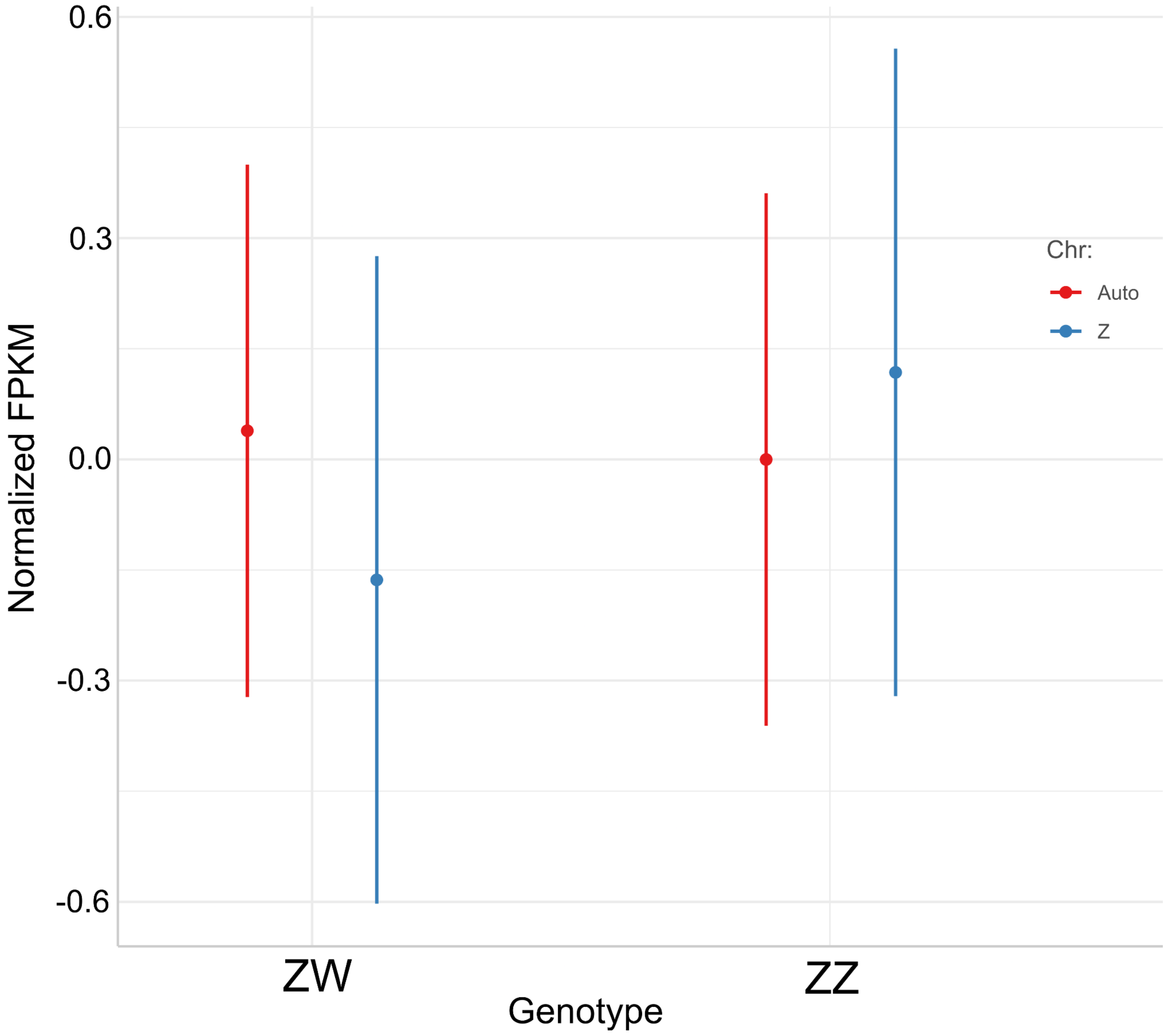

Supplemental Figure S4. Selection and dosage balance in Gila monster. Illustrating the effects of sexually concordant selection (x-axis) and sex-biased expression (y-axis) on dosage balance. For this figure, transcripts are marked as balanced or female-biased (blue) if the log_2 ratio of female to male expression is greater than -0.32 (equivalent to a raw ratio of approximately 0.8 or greater). On the x-axis, larger values indicate stronger sexually concordant selection. On the y-axis, more positive values are associated with a greater male bias, and more negative values are associated with a female bias.

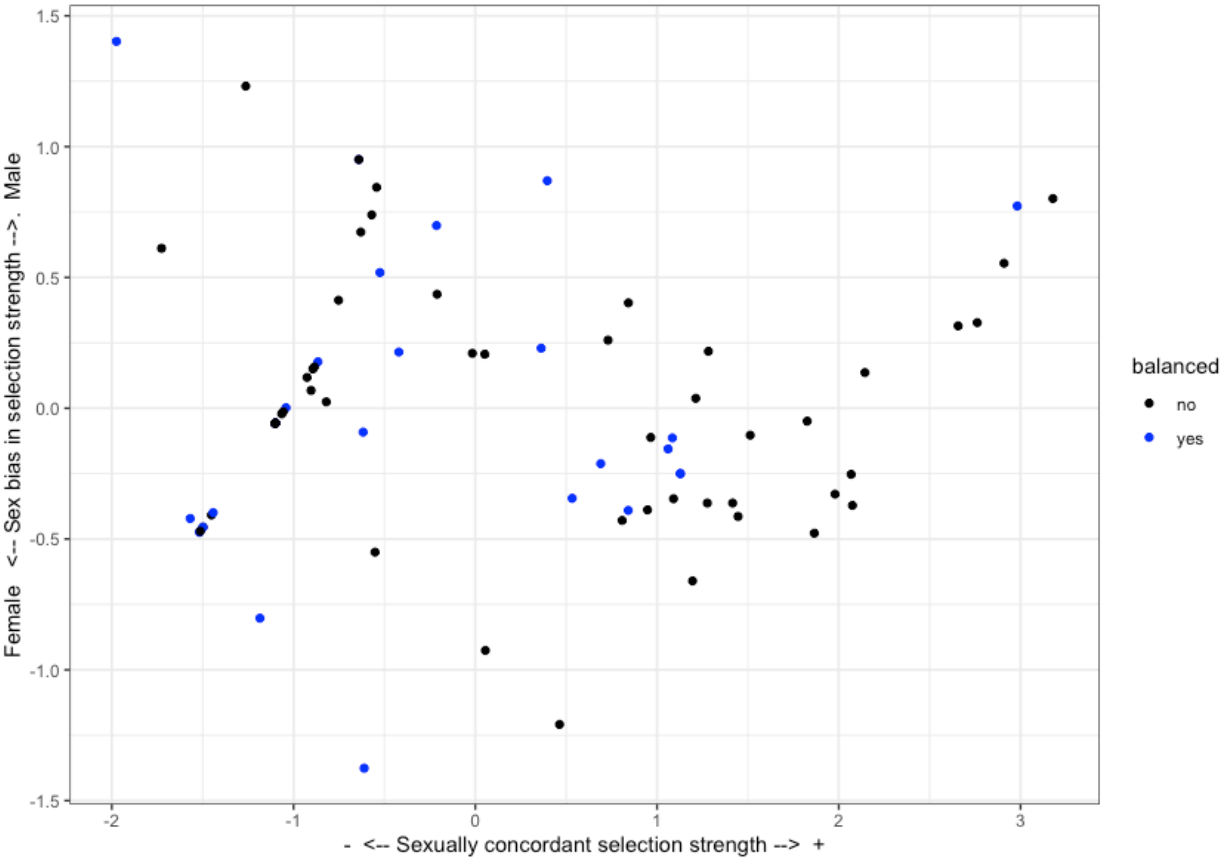

Supplement: evae018_Supplementary_Data [file evae018_supplementary_data.zip › Gila_monster_manuscript_GBEsubmission2_20231028_clean_supplement.pdf]
